# BCG coverage and barriers to BCG vaccination in Guinea-Bissau: an observational study

**DOI:** 10.1186/1471-2458-14-1037

**Published:** 2014-10-04

**Authors:** Sanne Marie Thysen, Stine Byberg, Marie Pedersen, Amabelia Rodrigues, Henrik Ravn, Cesario Martins, Christine Stabell Benn, Peter Aaby, Ane Bærent Fisker

**Affiliations:** Bandim Health Project, INDEPTH network, Apartado 861, 1004 Bissau Codex, Guinea-Bissau; Research Center for Vitamins and Vaccines (CVIVA), Statens Serum Intitut, Artillerivej 5, 2300 Copenhagen S, Denmark; Institute of Clinical Research, University of Southern Denmark/Odense University Hospital, 5000 Odense C, Denmark

**Keywords:** BCG, Coverage, Timeliness of vaccines, Implementation of the vaccination programme

## Abstract

**Background:**

BCG vaccination is recommended at birth in low-income countries, but vaccination is often delayed. Often 20-dose vials of BCG are not opened unless at least ten children are present for vaccination (“restricted vial-opening policy”). BCG coverage is usually reported as 12-month coverage, not disclosing the delay in vaccination. Several studies show that BCG at birth lowers neonatal mortality. We assessed BCG coverage at different ages and explored reasons for delay in BCG vaccination in rural Guinea-Bissau.

**Methods:**

Bandim Health Project (BHP) runs a health and demographic surveillance system covering women and their children in 182 randomly selected village clusters in rural Guinea-Bissau. BCG coverage was assessed for children born in 2010, when the restricted vial-opening policy was universally implemented, and in 2012–2013, where BHP provided BCG to all children at monthly visits in selected intervention regions. Factors associated with delayed BCG vaccination were evaluated using logistic regression models. Coverage between intervention and control regions were evaluated in log-binomial regression models providing prevalence ratios.

**Results:**

Among 3951 children born in 2010, vaccination status was assessed for 84%. BCG coverage by 1 week of age was 11%, 38% by 1 month, and 92% by 12 months. If BCG had been given at first contact with the health system, 1-week coverage would have been 35% and 1-month coverage 54%. When monthly visits were introduced in intervention regions, 1-month coverage was higher in intervention regions (88%) than in control regions (51%), the prevalence ratio being 1.74 (1.53-2.00). Several factors, including socioeconomic factors, were associated with delayed BCG vaccination in the 2010-birth cohort. When BCG was available at monthly visits these factors were no longer associated with delayed BCG vaccination, only region of residence was associated with delayed BCG vaccination.

**Conclusion:**

BCG coverage during the first months of life is low in Guinea-Bissau. Providing BCG at monthly vaccination visits removes the risk factors associated with delayed BCG vaccination.

**Electronic supplementary material:**

The online version of this article (doi:10.1186/1471-2458-14-1037) contains supplementary material, which is available to authorized users.

## Background

Bacillus Calmette Guérin (BCG) vaccine is recommended at birth to normal-birth-weight children in Guinea-Bissau. However, BCG vaccination is often delayed for several reasons, one of them being the “restrictive vial-opening policy”: BCG is a freeze-dried vaccine supplied in vials with 20 infant doses [1] and once reconstituted, the vaccine should only be used for a maximum of six hours. In Guinea-Bissau and other low-income countries [2, 3], the focus on not wasting vaccines [4] has led to a policy of not opening a BCG vial unless 10 children are present to be vaccinated [5]. Previous studies have shown that BCG has beneficial non-specific or heterologous effects, providing protection also against many non-tuberculosis causes of death [6–15]. In two randomised trials among low-birth-weight (<2500 g, LBW) neonates, BCG at birth compared with the usual delayed BCG, lowered neonatal mortality by 48% (95% CI: 18%-67%) [6, 7], the reduction being 58% (8%-81%) the first three days after vaccination [6, 7]. The rapidly occurring effect suggests that BCG stimulates the innate immune system. This is supported by recent immunological studies showing that BCG induces epigenetic changes which reprogram monocytes to increased pro-inflammatory responses against unrelated pathogens [16, 17].

Most infant deaths occur during the neonatal period, particularly in the first week of life [18] and thus any delays in BCG vaccination may have major consequences because children do not benefit from BCG when their mortality risk is highest. Hence, it is important to identify obstacles to early BCG vaccination, as this will help target interventions to lower the age at vaccination.

We assessed BCG coverage at different ages among children born in 2010 in rural Guinea-Bissau to identify factors associated with delayed BCG vaccination in a context with a restrictive vial-opening policy. In 2012, we implemented monthly visits and provided BCG vaccination to newborns in three intervention regions (Oio, Biombo and Cacheu) but not in six control regions. We evaluated how not adhering to the restrictive vial-opening policy affected BCG coverage and affected factors associated with delayed BCG vaccination.

## Methods

### Setting and study population

The study was conducted in the rural study area of the Bandim Health Project (BHP) in Guinea-Bissau. The BHP maintains a health and demographic surveillance system following 182 randomly selected clusters of 100 women and their children in rural Guinea-Bissau. The clusters were initially selected using the Expanded Programme on Immunizations (EPI) methodology for immunisation surveys sampling 20 clusters of 100 women in each of the eight larger health regions, and 10 and 12 clusters in the two smallest regions. Later two regions have been joined and rural Guinea-Bissau now has nine health regions; Oio, Biombo, Gabu, Cacheu, Bafata, Quinara, Tombali, Bubaque, and Bolama. All women of fertile age and children below the age of 5 years are followed through home visits every four to six months.

At the home visits women of fertile age are registered and information on ethnicity and schooling is collected. If a pregnancy is registered, a special form is completed collecting information on prenatal consultations and socioeconomic factors. For all newborn children information on date of birth, place of birth, and prenatal consultations is collected. At all visits the child’s vaccination card is inspected and vaccination dates are recorded. If the child has no vaccination card a vaccination card is provided from the BHP team.

### BCG vaccination possibilities

Children in Guinea-Bissau should receive BCG and oral polio vaccine (OPV) at birth, pentavalent vaccine (diphtheria-tetanus-pertussis-H. influenza type B-Hepatitis B vaccine) and OPV at 6, 10, and 14 weeks, and measles and yellow fever vaccines at 9 months. These vaccines are provided free of charge at the health centres as part of the national programme and during outreach to villages when additional funding is available. Due to the restricted vial-opening policy some vaccines are only provided once a week at health centres and only if there is a sufficient number of eligible children present.

Since 2007 the BHP teams visiting the surveyed villages in all regions have been accompanied by a nurse, who offered OPV, pentavalent vaccine, measles vaccine, and yellow fever vaccine to all children below the age of 1 year. The vaccines were supplied through the national programme and the BHP nurse had to follow the national policy. Hence she did not bring BCG as she would very rarely encounter sufficient eligible children in a village.

In 2012 the BHP increased the frequency of visits from four-six-monthly visits to monthly visits in three regions (Oio, Biombo and Cacheu). In these intervention regions BCG vaccination was offered to all children below the age of 1 year regardless of the number of children present, thus a BCG vial was opened for one child. In the remaining control regions national policy was followed and BCG was not offered during village visits.

### Information about reasons for delay of BCG vaccination

During the year from 1^st^ July 2012 to 30^th^ June 2013 mothers of BCG unvaccinated infants in all regions were interviewed on their BCG vaccination attempts/experiences during home visits. They were asked if they knew that their child was due to receive BCG and whether they had taken the child for vaccination. Mothers who reported to have sought vaccination were asked why the child had not been vaccinated. Mothers who had not sought BCG vaccination were interviewed about the reasons.

### Ethical approval

BHP’s HDSS which has been in place in Guinea-Bissau since 1978 and is conducted by request from the Guinean Ministry of Health. The current surveillance system in the rural areas has been approved by the National Ethics Committee in Guinea-Bissau and the Central Ethics Committee in Denmark. No separate ethical and consent approval was sought.

#### Statistical analyses

##### Assessing BCG coverage in the 2010-birth cohort

Standard estimates for vaccination coverage are usually based on vaccinations obtained by 12 months of age, assessed among children aged 12 to 23 months [19, 20]. In the 2010-birth cohort we assessed BCG coverage by 1 month of age in children aged 1–12 months at the time of the home visit, the coverage by 3 months of age in children aged 3–14 months, the coverage by 6 months in children aged 6–17 months, and the coverage by 12 months in children aged 12–23 months (Table 1). Since most neonatal deaths occur within the first week of life, we also assessed the 1-week coverage using vaccination status assessed within the 12 months after day 7. Vaccination status was determined at the first visit in the relevant time period at which the vaccination card was seen. Children who had lost their vaccination card or for whom the vaccination card was not seen were excluded from the analysis.

**Table 1:** **Coverage assessment methods for standard coverage estimates and coverage estimates in the 2010-birth cohort and the 2012 cohort**

	Standard coverage estimates	Coverage in 2010-birth cohort	Coverage in 2012 cohort
**Children**		Children born in 2010	Children aged 1, 3, 6, or 12 months of age, when visited from 1^st^ July 2012 to 30^th^ June 2013
**Assessment ages**	12 months	1 week, 1, 3, 6 and 12 months	1, 3, 6, and 12 months
**Vaccination status**	12-23 months	First visit with seen vaccination card within 12 months after assessment age	Visit with seen vaccination card in the month after the assessment age

Missed opportunities among BCG unvaccinated children were defined as contact with the health system, either being born at a health facility or having received other vaccines (based on the registered date of another vaccination on the vaccination card). We calculated the potential coverage if BCG had been given at the first contact with the health system.

### Assessing BCG coverage after implementation of monthly visits, 2012 cohort

In 2012 monthly visits were introduced in the intervention regions, whereas four-six-monthly visits continued in control regions. We considered a village as belonging to the intervention regions when there was less than 6 weeks between two subsequent visits. BCG coverage was assessed among all children visited from 1^st^ July 2012 to 30^th^ June 2013. Like in the 2010-birth cohort, we assessed BCG coverage at 1, 3, 6, and 12 months of age. To take into account that children in intervention regions with monthly visits had a larger possibility of having their card seen within a 12 months period compared with children in control regions with four-six-monthly visits, we considered only data collected in the month after 1, 3, 6, and 12 months, respectively (Table 1).

Comparisons of coverage between regions with different BCG provision strategies were evaluated in log-binomial regression models providing prevalence ratios (PR).

### Factors associated with delayed BCG vaccination

Factors associated with delayed BCG vaccination (vaccination after 1 month of age) were assessed in the 2010-birth cohort and after implementation of monthly visits separately for intervention and control regions in 2012/2013. This study focused on background factors assessed among the users of the vaccination services. The factors evaluated were: sex, birth place, antenatal care, region, type of roof, toilet, household possessions (radio, cell phone, and generator), ethnic group (Fula, Pepel, Balanta, Manjaco, and other), age of mother, and education of caretaker. All continuous variables were tested for linear relationship with BCG coverage by inspecting the BCG coverage in quintiles of the variable. Where inspection suggested a linear relationship, the quadratic value of the continuous variable was included in the model to assess departure from linearity. We tested all variables one by one in a simple model using logistic regression to calculate the odds ratio (OR) of being unvaccinated. As children were not individually sampled but selected for the study based on residence within a geographical cluster, we adjusted the standard error for cluster. In a larger multivariable model we included all factors associated with delayed BCG. In this large model we excluded ethnic group since ethnic group and region were highly correlated with more than 90% of the Pepels living in Biombo, and more than 75% of the Manjacos living in Cacheu. We chose to include region rather than ethnicity since region would be feasible to target through interventions.

## Results

### BCG coverage in the 2010-birth cohort

**Figure 1:**
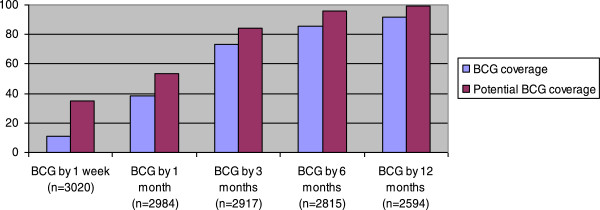
**Observed BCG coverage and potential BCG coverage by different ages.** Observed BCG coverage (blue) and potential BCG coverage if all vaccination opportunities had been used (purple). Bandim Health Project, Guinea-Bissau, 2010-birth cohort.

We assessed vaccination status at a visit within the first 2 years of life for 84% (3318/3951) of all children (Additional file 1). In the 2010-birth cohort, the BCG coverage by 1 week of age was 11% (327/3020) and 38% (1140/2984) by 1 month of age but increased to 92% (2385/2594) coverage by 12 months of age (Figure 1). If all children had received BCG vaccine at their first contact with the healthcare system, coverage would have been at least 35% by 1 week of age, 54% by 1 month of age and 99% by 12 months of age (Figure 1). The median age of BCG vaccination among children vaccinated within the first 12 months of life was 39 days. This could be reduced to 27 days if BCG vaccine had been given at first contact with the healthcare system.

### BCG coverage after implementation of monthly visits, 2012 cohort

**Figure 2:**
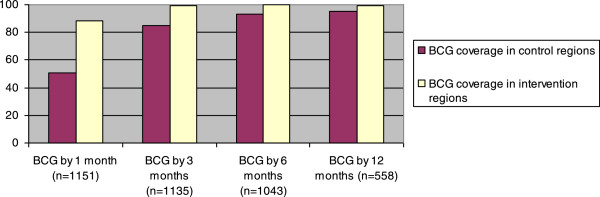
**Observed BCG coverage by different ages in intervention and control regions.** Bandim Health Project, Guinea-Bissau, 2012 cohort.

In the 2010-birth cohort, BCG coverage did not differ significantly between the regions which subsequently became intervention regions compared with the control regions (PR = 1.03 (0.85-1.24)) (data not shown). After implementation of monthly visits, we assessed BCG coverage among a total of 2812 children (Additional file 2). Coverage by 1 month of age was 88% (769/872) in intervention regions and 51% (141/279) in control regions (Figure 2), the PR being 1.74 (1.53-2.00). The 3-months coverage was 99% (769/776) in intervention regions and 85% (304/359) in control regions. By 12 months of age, it had increased to 99% (257/259) in intervention regions and 95% (284/299) in control regions. The potential 1-month coverage if BCG vaccine had been given at first contact with the healthcare system was 93% in intervention regions and 65% in control regions.

### Factors associated with delayed BCG vaccination in the 2010-birth cohort

A number of factors were associated with being BCG vaccinated by 1 month of age (Table 2). Region of residence was strongly associated; only 25% of children in Oio had received BCG compared with 60% on Bolama. Caretaker’s education was significantly associated with delayed BCG vaccination in both the univariate analysis (OR = 1.15 (1.10-1.20) per year of schooling), and the multivariable analysis (OR = 1.07 (1.02-1.12)). Previous contact with the health system was associated with higher BCG coverage in both the univariate and the multivariable analysis: Children born at health centres or hospitals were more likely to be BCG vaccinated (OR = 1.70 (1.26-2.30) and OR = 2.88 (2.06-4.01), respectively) than children born at home. Also, children of mothers who attended prenatal consultation were more likely to be BCG vaccinated (OR = 1.78 (1.23-2.57). The children born to mothers with better economic status reflected in possession of a latrine and possession of a cell phone had higher coverage; however, other socioeconomic factors were not significantly associated with coverage in the multivariable analysis. Maternal age was not associated with BCG coverage, and finally BCG coverage did not differ significantly for girls compared with boys (OR = 1.19 (0.99-1.43)) (Table 2).

**Table 2:** **Factors associated with BCG vaccination by 1 month of age**

	Total number of children	BCG by 1 month n (%)	OR of BCG vaccination^1^	Multivariable analysis OR (95%CI)^1^	P-value for the univariate/multivariable analyses
**Gender**					0.202/0.063
Male	1493	553 (37)	Ref	Ref	
Female	1491	587 (39)	1.10 (0.95-1.28)	1.19 (0.99-1.43)	
**Age of mother** ^**2**^					0.616/0.662
1.quartile (<21)	763	304 (40)	Ref	Ref	
2. quartile (21–24)	614	239 (39)	0.96 (0.77-1.21)	1.06 (0.80-1.40)	
3. quartile (25–30)	825	306 (37)	0.89 (0.71-1.11)	1.08 (0.82-1.43)	
4. quartile (>30)	745	275 (37)	0.88 (0.71-1.10)	1.18 (0.90-1.55)	
**Education of caretaker** ^**3,4**^					**<0.001/0.006**
None	1889	627 (33)	**1.15 (1.10-1.20)**	**1.07 (1.02-1.12)**	
1-4 years	648	286 (44)			
5+ years	347	185 (53)			
**Region** ^**5**^					**<0.001/0.007**
Oio	416	102 (25)	**0.37 (0.21-0.64)**	**0.39 (0.21-0.73)**	
Biombo	445	183 (41)	0.79 (0.50-1.24)	0.90 (0.60-1.35)	
Gabu	459	132 (29)	**0.45 (0.28-0.75)**	**0.50 (0.30-0.82)**	
Cacheu	582	274 (47)	Ref	Ref	
Bafata	370	122 (33)	**0.55 (0.31-0.98)**	**0.49 (0.27-0.89)**	
Quinara	277	143 (52)	1.20 (0.69-2.09)	1.07 (0.61-1.87)	
Tombali	277	105 (38)	0.69 (0.46-1.03)	0.84 (0.55-1.29)	
Bubaque	86	36 (42)	0.81 (0.46-1.42)	0.50 (0.24-1.01)	
Bolama	72	43 (60)	1.67 (0.98-2.84)	0.59 (0.33-1.04)	
**Ethnic group** ^**6**^					**<0.001**/NA
Balanta	739	265 (36)	1.27 (0.88-1.82)		
Mandinga/Fula	1116	342 (31)	Ref		
Manjaco	254	149 (59)	**3.21 (1.89-5.44)**		
Pepel	391	151 (39)	1.42 (0.92-2.20)		
Other	481	231 (48)	**2.09 (1.46-2.99)**		
**Contact with the health system**					
***Birth place*** ^***7***^					**<0.001/<0.001**
At home	1888	576 (31)	Ref	Ref	
Healthcare centre	527	263 (50)	**2.27 (1.68-3.07)**	**1.70 (1.26-2.30)**	
Hospital	380	228 (60)	**3.42 (2.61-4.48)**	**2.88 (2.06-4.01)**	
Other	29	4 (14)	0.36 (0.13-1.02)	0.43 (0.16-1.18)	
***Prenatal consultations*** ^***8***^					**<0.001/0.002**
Yes	2313	942 (41)	**2.88 (2.06-4.02)**	**1.78 (1.23-2.57)**	
No	301	58 (19)	Ref	Ref	
**Socioeconomics**					
***Type of roof*** ^***9***^					**<0.001**/0.324
Straw	1427	482 (34)	Ref	Ref	
Hard	1522	649 (43)	**1.46 (1.17-1.81)**	1.13 (0.89-1.43)	
***Toilet*** ^***10***^					**0.001/0.005**
None	903	286 (32)	Ref	Ref	
Latrine/ toilet in house	2037	839 (41)	**1.51 (1.18-1.94)**	**1.54 (1.14-2.08)**	
**Household possessions**					
***Cell phone*** ^***11***^					**<0.001/0.025**
Yes	1339	571 (43)	**1.41 (1.20-1.64)**	**1.24 (1.03-1.49)**	
No	1575	545 (35)	Ref	Ref	
***Radio*** ^***12***^					0.211/0.192
Yes	2104	825 (39)	1.13 (0.94-1.36)	0.87 (0.71-1.07)	
No	810	295 (36)	Ref	Ref	
***Generator*** ^***13***^					**0.025**/0.563
Yes	187	88 (47)	**1.47 (1.05-2.07)**	1.14 (0.73-1.80)	
No	2753	1036 (38)	Ref	Ref	

Bandim Health Project, Guinea-Bissau, 2010-birth cohort.

^1^Standard error adjusted for clustering by robust variance estimates.

^2^Numbers do not add up due to some not living with their mother.

^3^Education of caretaker per year’s schooling; linear.

^4^100 had missing information on years of schooling.

^5^When including ethnic group rather than region in the final model the estimates changed less than 10% for all parameters assessed.

^6^3 had missing information on ethnic group.

^7^160 had missing information on place of birth.

^8^370 had missing information on prenatal consultations.

^9^35 had missing information on type of roof.

^10^44 had missing information on possession of a latrine.

^11^70 had missing information on possession of a cell phone.

^12^70 had missing information on possession of a radio.

^13^44 had missing information on possession of a generator.

Note: significant (p < 0.05) findings in bold.

The risk factor analysis for BCG coverage by 3, 6, and 12 months identified the same factors but most associations were weaker. Prenatal consultation was significantly associated with BCG coverage at all ages (data not shown).

### Factors associated with delayed BCG vaccination after implementation of monthly visits, 2012 cohort

After implementation of monthly visits, factors associated with delayed BCG vaccination were studied among the 2812 children who had a vaccination status assessed by 1, 3, 6, or 12 months of age. In the control regions (n = 1147) the factors strongest associated with being BCG vaccinated by 1 month of age in the 2012 cohort were region, contact with the healthcare system (being born at a hospital; OR = 1.81 (1.20-2.73)), and living in a house with hard roof (OR = 1.74 (1.30-2.32)). Other factors were significantly associated with delayed BCG in the univariate analysis, but not when adjusted for the other factors (Table 3). When the visit frequency was increased to monthly visits (n = 1665) socioeconomic factors and contact with the healthcare system were no longer significantly associated with BCG coverage, only region was significantly associated with BCG coverage in the multivariable analysis (Table 3).

**Table 3:** **Factors associated with BCG vaccination by 1 month of age after implementation of monthly visits in intervention regions**

	Intervention regions				Control regions			
	BCG by 1 month n (%)	OR of early BCG vaccination^1^	Multivariable analysis OR (95% CI)^1^	P-value for the univariate/multivariable analyses	BCG by 1 month n (%)	OR of early BCG vaccination^1^	Multivariable analysis OR (95% CI)^1^	P-value for the univariate/multivariable analyses
**Gender** ^**2**^				0.492 / 0.662				0.099/0.070
Male	658 (77)	Ref	Ref		284 (50)	Ref	Ref	
Female	620 (76)	0.93 (0.75-1.15)	1.05 (0.84-1.32)		260 (45)	0.82 (0.65-1.04)	0.78 (0.59-1.02)	
**Age of mother** ^**3**^				0.582 / 0.615				0.998/0.979
1.quartile (<21)	339 (78)	Ref	Ref		125 (47)	Ref	Ref	
2. quartile (21–26)	244 (78)	1.01 (0.68-1.49)	0.95 (0.59-1.53)		113 (47)	1.00 (0.69-1.47)	1.09 (0.69-1.71)	
3. quartile (27–31)	357 (76)	0.90 (0.64-1.27)	0.86 (0.60-1.25)		153 (48)	1.01 (0.71-1.45)	1.03 (0.65-1.63)	
4. quartile (>31)	335 (75)	0.83 (0.59-1.17)	0.80 (0.53-1.22)		153 (48)	1.03 (0.75-1.40)	1.07 (0.72-1.59)	
**Education of caretaker** ^**4, 5**^				**0.003** / 0.377				**0.018**/0.771
None	607 (73)	**1.08 (1.03-1.14)**	1.03 (0.96-1.10)		317 (46)	**1.07 (1.01-1.13)**	0.99 (0.92-1.07)	
1-4 years	323 (79)				118 (47)			
5+ years	270 (83)				84 (59)			
**Region** ^**6**^				**<0.001 / 0.002**				**0.003**/0.161
Oio	271 (70)	**0.67 (0.46-0.95)**	0.70 (0.49-1.02)		81 (40)	**0.49 (0.31-0.77)**	0.67 (0.40-1.09)	
Biombo	528 (81)	1.21 (0.87-1.68)	1.34 (0.91-1.98)		42 (47)	0.67 (0.41-1.11)	0.92 (0.56-1.51)	
Gabu		NA	NA		83 (40)	**0.50 (0.31-0.82)**	0.63 (0.36-1.10)	
Cacheu	479 (77)	Ref	Ref		148 (57)	Ref	Ref	
Bafata		NA	NA		42 (39)	**0.48 (0.27-0.88)**	**0.54 (0.30-0.98)**	
Quinara		NA	NA		112 (57)	1.01 (0.59-1.73)	1.18 (0.68-2.04)	
Tombali		NA	NA		24 (37)	0.44 (0.17-1.15)	0.69 (0.26-1.89)	
Bubaque		NA	NA		6 (60)	1.13 (0.43-2.95)	1.61 (0.43-6.07)	
Bolama		NA	NA		7 (70)	1.75 (0.52-5.94)	1.82 (0.30-10.99)	
**Ethnic group** ^**7**^				0.109 / NA				**<0.001**/NA
Balanta	401 (73)	Ref			124 (41)	Ref		
Mandinga/Fula	194 (76)	1.18 (0.81-1.73)			175 (39)	0.92 (0.66-1.28)		
Manjaco	140 (79)	1.37 (0.79-2.38)			64 (67)	**2.84 (1.49-5.40)**		
Pepel	426 (81)	**1.60 (1.12-2.27)**			35 (48)	1.31 (0.80-2.13)		
Other	115 (74)	1.07 (0.66-1.73)			147 (63)	**2.45 (1.65-3.65)**		
**Contact with the health system**								
***Birth place*** ^***8***^				0.369 / 0.515				**<0.001/<0.001**
At home	793 (76)	Ref	Ref		337 (43)	Ref	Ref	
Healthcare centre	255 (81)	1.38 (0.95-1.99)	1.30 (0.83-2.03)		82 (53)	**1.48 (1.01-2.16)**	1.15 (0.76-1.74)	
Hospital	192 (78)	1.17 (0.83-1.66)	0.96 (0.64-1.44)		106 (62)	**2.18 (1.54-3.08)**	**1.81 (1.20-2.73)**	
***Prenatal consultations*** ^***9***^				0.410 / 0.504				0.057/0.558
Yes	1093 (79)	1.16 (0.81-1.66)	0.86 (0.55-1.34)		460 (49)	1.55 (0.99-2.44)	1.20 (0.71-2.05)	
No	124 (76)	Ref	Ref		42 (38)	Ref	Ref	
**Socioeconomics**								
***Type of roof*** ^***10***^				**0.048** / 0.128				**<0.001/<0.001**
Straw	493 (74)	Ref	Ref		151 (37)	Ref	Ref	
Hard	777 (79)	**1.35 (1.00-1.83)**	1.28 (0.93-1.77)		386 (54)	**1.97 (1.50-2.58)**	**1.74 (1.30-2.32)**	
***Toilet*** ^***11***^				**0.039** / 0.188				0.136/0.706
None	445 (74)	Ref	Ref		100 (44)	Ref	Ref	
Latrine/ toilet in house	819 (79)	**1.32 (1.01-1.73)**	1.24 (0.90-1.71)		439 (49)	1.23 (0.94-1.62)	0.93 (0.67-1.30)	
**Household possessions**								
***Cell phone*** ^***12***^				**0.011** / 0.136				**0.009**/0.347
Yes	747 (80)	**1.38 (1.08-1.78)**	1.25 (0.93-1.66)		311 (51)	**1.39 (1.08-1.79)**	1.15 (0.84-1.55)	
No	501 (74)	Ref	Ref		221 (43)	Ref	Ref	
***Radio*** ^***13***^				0.183 / 0.303				0.159/0.841
Yes	958 (78)	1.21 (0.91-1.59)	1.17 (0.87-1.56)		422 (49)	1.24 (0.92-1.67)	1.05 (0.75-1.48)	
No	295 (74)	Ref	Ref		109 (43)	Ref	Ref	
***Generator*** ^***14***^				**0.020** / 0.083				0.234/0.636
Yes	149 (83)	**1.51 (1.07-2.13)**	1.46 (0.95-2.24)		53 (53)	1.27 (0.86-1.87)	0.89 (0.54-1.47)	
No	1115 (76)	Ref	Ref		485 (47)	Ref	Ref	

Bandim Health Project, Guinea-Bissau, 2012 cohort.

^1^Standard error adjusted for clustering by robust variance estimates.

^2^1 had missing information on gender.

^3^Numbers do not add up due to some not living with their mother.

^4^Education of caretaker per year’s schooling; linear.

^5^157 had missing information on years of schooling.

^6^When including ethnic group rather than region in the final model the estimates changed less than 10% for all parameters assessed.

^7^3 had missing information on ethnic group.

^8^56 had missing information on place of birth, 26 were born elsewhere and have been excluded due to small numbers.

^9^202 had missing information on prenatal consultations.

^10^33 had missing information on type of roof.

^11^36 had missing information on possession of a latrine.

^12^71 had missing information on possession of a cell phone.

^13^58 had missing information on possession of a radio.

^14^37 had missing information on possession of a generator.

Note: significant (p < 0.05) findings in bold.

### Information about reasons for not being BCG vaccinated

The year following implementation of monthly visits in intervention regions, 1470 interviews were conducted with mothers of BCG unvaccinated children from all regions. Among the mothers 229 (16%) reported to have sought vaccination for their child, 135 (59%) recalled to be told to return another day to get the vaccine, and 76 (33%) had received other vaccines (Additional file 3). Among the 1239 mothers, who reported not to have sought vaccination, 760 (61%) reported that their main reason was lack of money whereas 481 (39%) said that the distance to the vaccination post kept them from seeking vaccination (Additional file 3).

## Discussion

### Main findings

In 2010, BCG coverage by 1 week of age was only 11% in rural Guinea-Bissau. By 1 month of age the coverage was 38%, increasing to 73% by 3 months of age and 92% by 12 months of age. Contact with the health system was one of the main factors associated with BCG vaccination, but socioeconomic factors also played a role. When monthly visits were introduced in intervention regions and BCG was available for all children the inequity was reduced and the 1-month BCG coverage was 88% compared with 51% in control regions.

### Strengths and weaknesses

A major strength of this study is the set-up in the form of the health and demographic surveillance system covering a representative part of the population in rural Guinea-Bissau. Data was collected through frequent home visits by experienced field workers. Weaknesses include that children who died before the assessment age did not enter the coverage analysis; however, children dying before 12 months of age usually do not enter the standard coverage estimation either. Also it should be noted that the vaccination coverage was estimated using slightly different approaches in the 2010-birth cohort and the 2012 cohort. However, we do not directly compare coverage between the cohorts, but only compare coverage between intervention and control regions within the 2012 cohort. Information on reasons for not being vaccinated was collected based on the mothers’ recall.

### Consistency with other studies

Vaccination coverage is usually reported by 12 months of age. We found 92% BCG coverage by 12 months in 2010, which corroborates the 94% coverage from 2009 reported by WHO [21]. Others have found that the median vaccination coverage across 31 low- and middle-income countries was 98% and ranged from 56% to 100% [19].

We found a much lower coverage by 1 and 3 months, which concurs with reports of a median coverage across the 31 countries of 65% by age of 4.3 weeks, ranging from 15% to 97% [19]. This supports the need for assessing BCG coverage at earlier ages to disclose the delay in BCG vaccination.

Other studies [22–24] defined timely BCG vaccination as vaccinated before 8 weeks of age. The percentage of timely vaccinated children ranged from 69% vaccinated [22] in a large survey in 45 low-income and middle-income countries, to 99% [24] in a study from three areas in South Africa. This still does not fully disclose the poor coverage in the neonatal period, with only 49% coverage by 4 weeks [22], quite similar to the 1-month coverage of 38% in the present study.

We found that giving birth at a hospital or health centre increased the likelihood of being BCG vaccinated. Similarly, a study from South Africa found that birth at a health facility reduced the risk of being unvaccinated by 47% (26%-42%) [24]. In Ethiopia there was also higher BCG coverage for those born at a health facility [25]. In Guinea-Bissau it is not general practice that children born at a hospital or health centre are vaccinated before they leave the health facility, however, they are often told to return to the health centre for vaccination.

It has previously been reported from South Africa [24] and 31 low- and middle-income countries [19] that low socioeconomic status was related to delay in BCG vaccination. We found a similar tendency, especially during the first month of life. Importantly, this inequity in getting BCG vaccinated was no longer apparent when we provided BCG at monthly village visits.

### Interpretation and implications

WHO vaccination coverage estimates are reported as the coverage by 12 months of age not taking into account the timeliness of vaccines received. This does not disclose delays in administration of the BCG vaccine. It has been shown that BCG vaccination can reduce neonatal mortality by 48% in LBW children when administered at birth [6]. When donors only ask for BCG coverage by 12 months of age, there is no incentive to provide BCG in the neonatal period. Therefore the 1-month coverage or the median age at vaccination would be better indicators of BCG coverage and its likely effect on child survival.

Obstacles to timely BCG were identified through the interview with mothers of unvaccinated children. Among the mothers having sought vaccination but had not obtained BCG more than half the mothers recalled being told to return another day. We speculate that the restricted vial-opening policy is one of the main obstacles to early BCG vaccination but the information disclosed to the mothers does not allow any final conclusion. Among the mothers who had not sought vaccination not having money and distance to health facility were the main obstacles to taking their child for vaccination. Routine childhood vaccinations are provided free of charge in Guinea-Bissau, but health workers charge fees (~1$) for vaccination cards.

Twenty-five percent of BCG unvaccinated children had been in contact with a health facility by 1 month of age. The potential coverage by 1 month of age was 54% in 2010, if all children had been BCG vaccinated at first contact with the health system. Monthly village visits with BCG vaccination for all children significantly increased BCG vaccination coverage, especially at early ages, and would provide a very efficient tool for increasing especially coverage among the youngest children.

## Conclusions

Our study showed a large delay in BCG vaccination in Guinea-Bissau with less than half of the children being BCG vaccinated by 1 month and only 11% being BCG vaccinated by 1 week of age. Our risk factor analysis identified many factors associated with delay of BCG vaccination, including a number of socioeconomic factors, but these factors were no longer associated with delayed BCG vaccination when BCG-vaccination became available to all children at monthly visits.

## Electronic supplementary material

Additional file 1: **Flowchart. Bandim Health Project, Guinea-Bissau, 2010 rural birth cohort.** (PDF 19 KB)

Additional file 2: **Flowchart. Bandim Health Project, Guinea-Bissau, 2012 rural cohort.** (PDF 19 KB)

Additional file 3: **Reasons for not being BCG vaccinated. Bandim Health project, Guinea-Bissau, BCG unvaccinated children when met by the BHP team in 2012.** (PDF 4 KB)

## Abbreviations

BCG: Bacillus Calmette-Guérin vaccine
BHP: Bandim Health Project
CI: Confidence interval
LBW: Low-birth-weight
OPV: Oral polio vaccine
OR: Odds ratio
PR: Prevalence ratio
WHO: World Health Organization.
