# Supplementary figures and images for: BCG coverage and barriers to BCG vaccination in Guinea-Bissau: an observational study
Source: BMC Public Health. 2014 Oct 4;14:1037. doi: 10.1186/1471-2458-14-1037 (PMC4195857; doi:10.1186/1471-2458-14-1037)

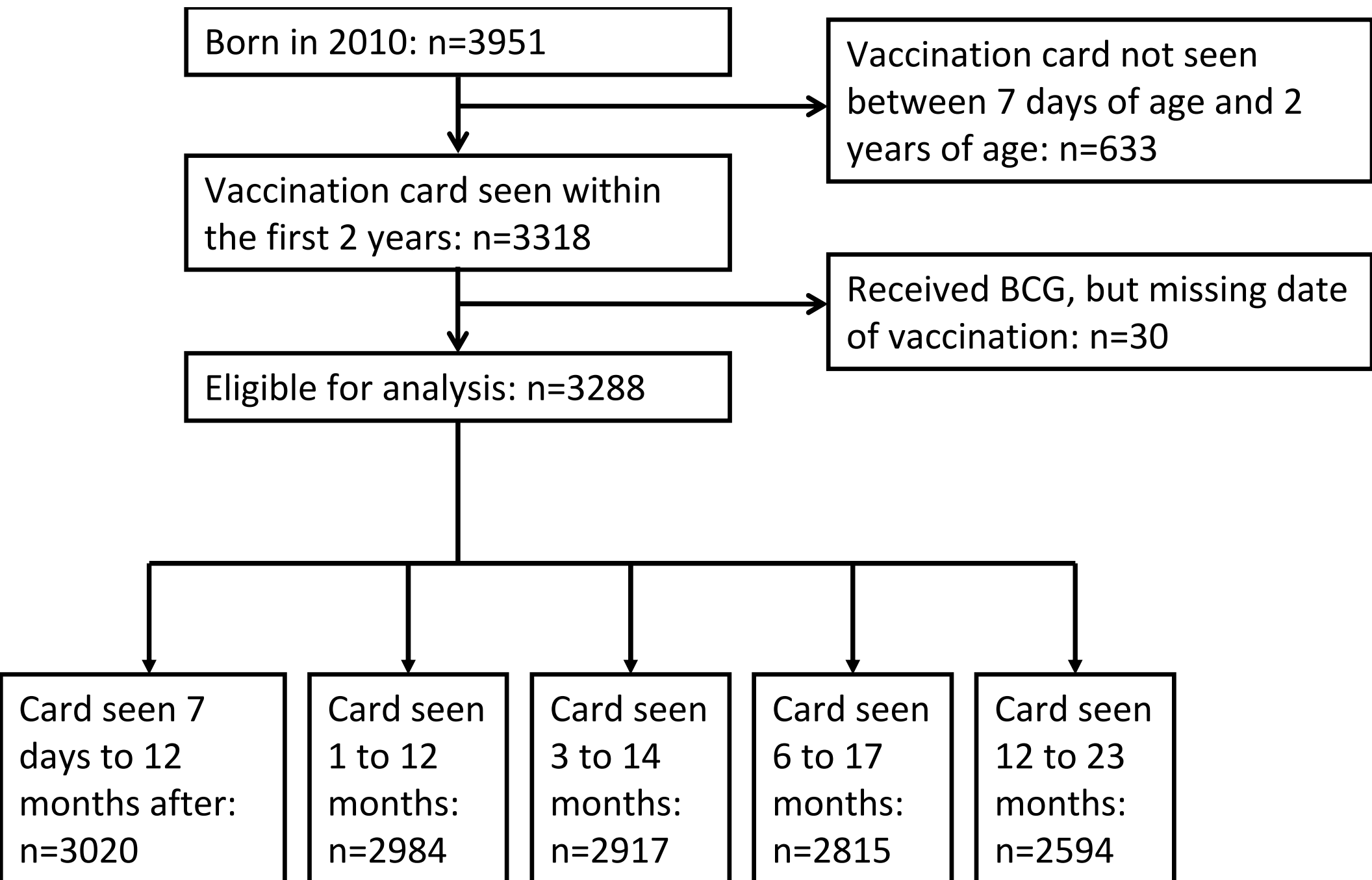

Supplement: Supplementary file 1 — Additional file 1: Flowchart. Bandim Health Project, Guinea-Bissau, 2010 rural birth cohort. (PDF 19 KB) [file 12889_2014_7145_MOESM1_ESM.pdf]

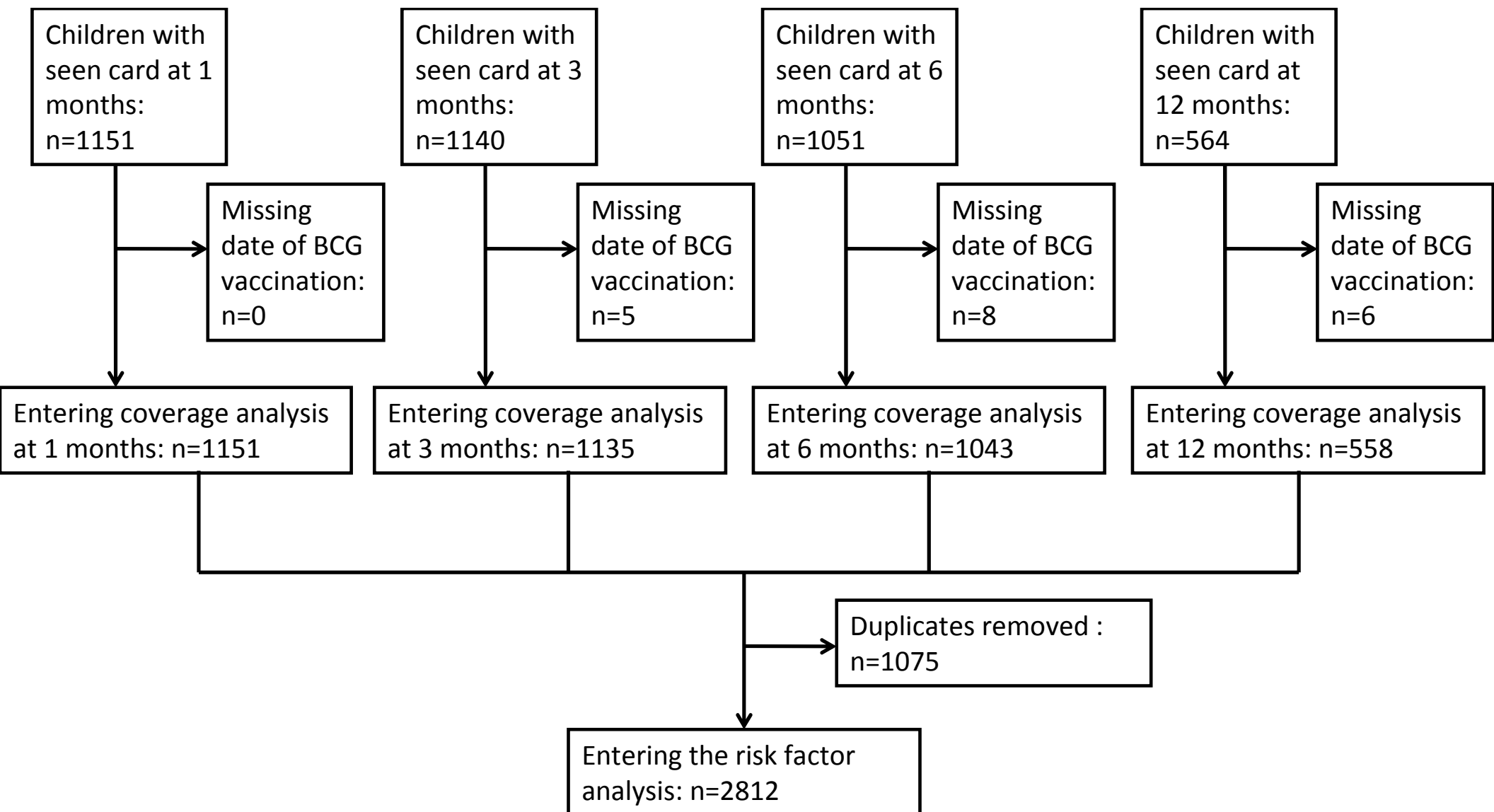

Supplement: Supplementary file 2 — Additional file 2: Flowchart. Bandim Health Project, Guinea-Bissau, 2012 rural cohort. (PDF 19 KB) [file 12889_2014_7145_MOESM2_ESM.pdf]
