# Supplementary material for: BCG coverage and barriers to BCG vaccination in Guinea-Bissau: an observational study
Source: BMC Public Health. 2014 Oct 4;14:1037. doi: 10.1186/1471-2458-14-1037 (PMC4195857; doi:10.1186/1471-2458-14-1037)
Supplement: Supplementary file 3 — Additional file 3: Reasons for not being BCG vaccinated. Bandim Health project, Guinea-Bissau, BCG unvaccinated children when met by the BHP team in 2012. (PDF 4 KB) [file 12889_2014_7145_MOESM3_ESM.pdf]

	Number[1]
Number	1470
Median age (days) at the time of the interview (Inter quartile range)	56 (24-111)
Reported to have sought vaccination[2]	229 (16%)
Recalled being told to return another day	135 (59%)
Received other vaccinations	76 (33%)
Reported not to have sought vaccination[3]	1239 (84%)
Received other vaccinations	39 (3%)
Knew the child was due to be vaccinated	1123 (91%)
Lack of money	760 (61%)
Distance to vaccination place too long	481 (39%)
Waiting for outreach services in the village.	396 (32%)

[1] Number (%) unless reported otherwise
[2] Children can be included in more than one category
[3] Mothers can report several reasons not having sought vaccination
